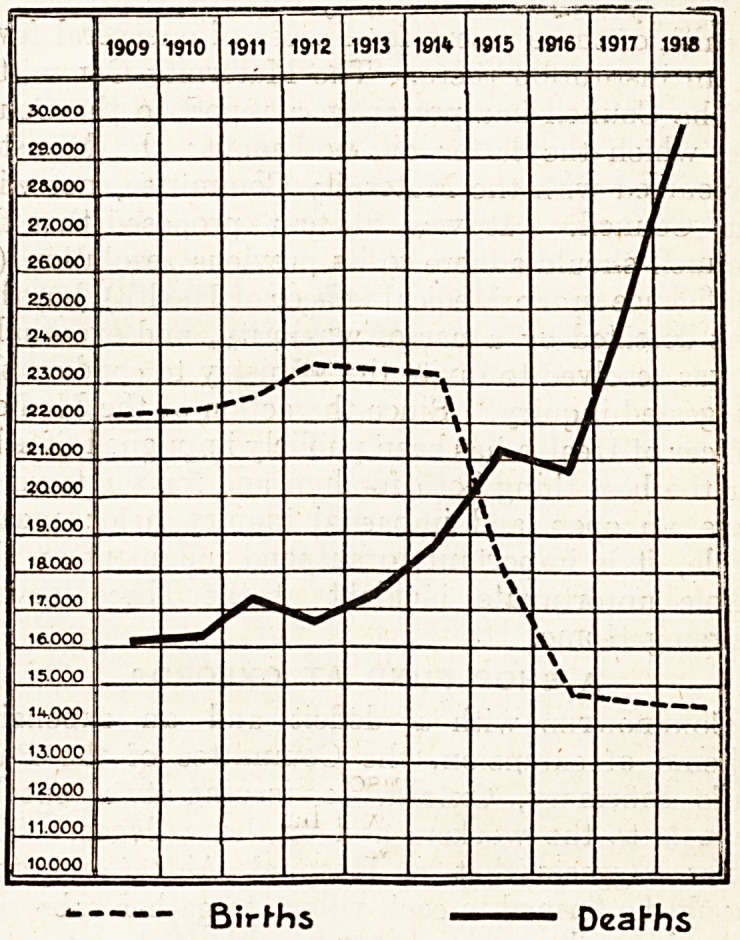# Hospital and Institutional News

**Published:** 1920-08-14

**Authors:** 


### HOSPITAL AND INSTITUTIONAL NEWS.

### LORD ALLENBY'S APPEAL FOR A BRITISH HOSPITAL IN CAIRO.

Field-Marshal Lord Allenby is appealing to the British Public for funds towards the £150,000 needed to found and endow a hospital in Cairo for British patients, staffed by British doctors and nurses. At the present time, Lord Allenby points out, there exist two small institutions which are attempting to cope with the very considerable amount of this work which requires to be done: the Anglo-American Hospital founded by Lord Cromer in 1903, "which has made a gallant struggle for success, handicapped by insufficient funds, a remote and inaccessible situation, and inadequate accommodation"; and the Victoria Deaconess Hospital, under the joint management of the American, British, German, and Swiss communities, which does for the poorer classes what the Anglo-American does for those of less indigent circumstances. His lordship states that a committee has reported that addition to the present Anglo-American Hospital and the unification of this institution with the Deaconess Hospital are alike impossible; and he appeals for a new institution on a national basis and an adequate scale. Ample provision for the poorer classes and the appointment of a Resident Medical Officer are part of the project, which apparently involves the supersession of the Anglo-American Hospital. We trust that an effort will be made to obtain American as well as British support, and to accept American patients, though Lord Allenby does not specifically state that this will be the case; and we trust that the response to his appeal will be prompt and generous.

### THE MEMORIAL TO SIR WILLIAM OSLER.

A General Committee has now been formed to raise funds for the memorial to Sir William Osler, which was decided on at the public meeting at Oxford on March 6 (see The Hospital, March 13, page 561). The memorial is to be the erection of an Osler Institute of General Pathology and Preventive Medicine. The patrons of the appeal for funds are the Prince of Wales, the U.S. Ambassador in England, the British Ambassador at Washington, the High Commissioner for Canada, and the Chancellor of the University of Oxford (Earl Curzon of Kedleston). The list of the General Committee is a long one and includes names distinguished in the medical profession, the universities, the official world, literature, and science. The Chairman of the Executive Committee is the new Bishop of Ripon (Very Rev. Sir T. B. Strong, formerly Dean of Christ Church), its honorary treasurer is Mr. A. P. Dodds-Parker, surgeon to the Radcliffe Infirmary, and the Honorary Secretary, Dr. J. A. Gunn, Professor of Pharmacology in the University of Oxford. Subscriptions should be sent to the Treasurer, Mr. A. P. Dodds-Parker, Holywell, Oxford, or to the National Provincial and Union Bank of England, 91 High Street, Oxford.

### EMPLOYERS' SUBSCRIPTIONS AND SMALLER HOSPITALS.

So far as we can gather, there is some confusion concerning the meaning of the employers' subscription funds which have been successfully started at Leeds and other centres, and more lately at Sheffield. These big industrial centres understand the term well enough, but the men at the smaller hospitals, which can benefit from these funds also, appear to think that an employers' subscription list and an employers' subscription fund are the same thing, and they go on as before accordingly. The difference, of course, is that, in the case of the funds, the subscriptions of the employers are related to the number of their employees. Almost every small hospital has employers among its subscribers, but the recent plan is to methodise their subscriptions by basing them upon the number of their workpeople. The plan has the advantage that the entire group of local employers can be approached, and that when they have agreed to it their workpeople will generally agree to weekly contributions, which are almost as rare in smaller places as they are common in the big ones. Let no one, therefore, whose hospital has employers among its subscribers think the plan is already adopted. It involves the standardisation of their subscriptions by giving them a *pro rata* basis adopted by all the firms and their staffs in the neighbourhood.

### A PRIVATE DONOR OF A WAR MEMORIAL.

Leicester is a public-spirited county in hospital matters, and a fine example has been set by Colonel R. Dagliesh, C.B., who has offered to build, at his own cost, the cottage hospital which is to be the war memorial of Melton Mowbray. A building already purchased is in use as a temporary hospital, and the idea is to build a hospital in the grounds and to use the present house as an administrative block. The plans prepared by Messrs. Shelbourn are those which Col. Dagliesh proposes to carry out. Without his aid, the scheme might have miscarried through lack of support, and this fact lends value to the simple conditions which the donor lays down. He wants the funds already subscribed or promised towards the cost of construction, other than those spent on the site and adaptation, to be invested as the beginning of an endowment fund, and the constitution of the Committee modified to ensure the inclusion as ex-officio members of a certain number of representatives of local bodies. The Hospital Committee, of which Mr. S. H. Gardner, J.P., is Chairman, have accepted this offer with gratitude, and surely all must feel that if Colonel Dagliesh builds the local war memorial hospital, the least they can do is to maintain it.

### MR. LLOYD GEORGE AND SALFORD HOSPITAL.

We are glad to announce that the special committee in charge of the scheme for the Salford War Memorial has decided to devote the money subscribed to the Salford Royal Hospital. It amounts to £26,000, and will be used to endow a ward and to place commemorative tablets in the ward itself and in the entrance hall of the hospital. In September Mr. Lloyd George will receive the freedom of the borough, and it is hoped that his visit will include a personal attendance at the memorial ceremony.

### A FERTILE PRESIDENT.

The retiring President of the East Suffolk and Ipswich Hospital, Mr. E. G. Pretyman, M.P., has taken an active part in raising the hospital's income. His recent suggestion of organising collections in the rural districts from the schools and agriculturists was described in these columns a few weeks ago, and the other day, when the deficit, double that of a year ago, of £10,600 was the subject of anxious discussion, and four representatives of the workers were added to the Board, Mr. Pretyman said that he took a fatherly interest in the satisfactory growth of the weekly collections, because he had originally suggested the idea after observing its workings at Grimsby. Mr. J. D. Cobbold recognised Mr. Pretyman's work by saying that his services had been so valuable that they all hoped Mr. Pretyman would one day be their President again. The new President is Lord Cranworth, and if he can consolidate the support of the county as that of the town has been, the hospital, which is taking twice the number of patients that it had in 1913, should soon be free of its deficit.

### GLASS OXYGEN ROOMS.

We note that popular attention has been lately drawn to an undoubtedly valuable new method of treating certain pulmonary conditions. This is the so-called "oxygen ward," a glass chamber of some 1,000 cubic feet capacity, capable of holding three beds, and which first came into existence during the war as a method of treating "gassed" patients. The idea is to maintain an atmosphere within the chamber containing about 30 to 50 per cent. of oxygen, the remainder being air, and a series of "air locks" allow entry and exit of patients and attendants and the provision of food. Several experimental units have been erected in this country, and we know of at least one Metropolitan hospital which has installed an oxygen chamber in one of its medical wards. Patients have been allowed to remain in the "ward" for spells of twenty hours at a time, and there is no doubt that men in both the acute and chronic bronchitic states have greatly benefited by the use of an enriched oxygen atmosphere. Further reports on the results obtained with the usual acute pulmonary cases seen in civilian practice will be awaited with considerable interest. At present, of course, the cost of such treatment is necessarily high, but the prospects are good that results obtained will justify the outlay involved.

### ANIMAL ASTHMAS.

Considerable interest attaches to a paper published in a recent issue of the *Lancet*, and containing a reprint of an address delivered before the Royal Society of Medicine by Dr. J. Freeman, the Director of the Department of Clinical Bacteriology at St. Mary's Hospital, London. There has in the past been much speculation, popular and professional, concerning "horse asthma," "hay fever," "cat asthma," and other baffling afflictions of the same nature, and Dr. Freeman has rendered a valuable service in correlating the known etiology of these diseases on what is at least the beginning of a sound medical basis. He proves conclusively that these "asthmas" are specific for the individual and the irritant substance concerned, and he brings together good evidence for his suggestion that the conditions are of a definitely hereditary nature. More attractive still is the outline of possible diagnostic means which may be employed, and to spend some time in carefully reading his address will well repay the majority of practising physicians.

### BEDFORD'S CONSTANT PROGRESS.

The new Convalescent Home of the Bedford County Hospital at "Homewood," Aspley Heath, was opened on July 31 by the Lord-Lieutenant of Bedfordshire, Mr. S. H. Whitbread, C.B. It is situated among the Woburn pinewoods, over 400 feet above the sea, on light, sandy soil, with garden and grounds of over four acres. The house, which is equipped for ten male and ten female convalescents, is provided with central heating and electric light plant. The cost of purchase, alterations, and equipment was about £8,000, and the Home was opened free of debt. The next item in the "forward" programme of the Bedford County Hospital is the provision in the near future of wards for paying patients in a house adjoining the hospital, which will be made available through the generosity of the Hospital's President, Mr. S. H. Whitbread. Bedford County is fortunate in its hospital and its chairman, Mr. J. Arnold Whitchurch, is entitled to congratulation on the spirit of progress which characterises the conduct of its affairs.

### A PROGRESSIVE INCOME AT SWANSEA.

The Swansea Hospital has made good progress financially of late and, if Mr. Roger Beck's expectations are realised, its income should advance considerably before long. Not only has the waiting list been reduced by seventy-five, but subscriptions and donations are £2,000 more than in the corresponding period a year ago, and contributions from local workmen have nearly doubled and at present amount to well over £8,000 for the half-year. Mr. Beck, in view of the promises which he has received, hopes shortly for £20,000 from this source. In fact for the half-year the total revenue is £23,66[illegible] against £10,000 odd a year ago. This is excellent progress, although £3,000 has been added to the debt, because little more than £1,000 has been received from legacies. The immediate need [illegible] Swansea is for a few generous men to pay [illegible] arrears, since such excellent progress has been made toward meeting the cost of maintenance. Has Mr. Beck any plans by which this can be done?

### A CAREFULLY PREPARED APPEAL.

A good beginning has been made with the appeal for £50,000 to extend the Chesterfield and North Derbyshire Hospital. Mr. E. C. Barnes, chairman of the Board of Governors, reported to the inaugural public meeting that to obtain the £6,000 allotted by the Red Cross Society the contract for the extensions should be signed by August 31. He describes the present out-patient department as a disgrace, and the extensions will properly provide for the group of departments of which the out-patients' section consisted. The plan is to build it on the site of the house and grounds lately given by Mr. Eastwood. For the nursing and domestic staff the presented building, Holywell House, will be used to avoid the expense of a new building. The shorter hours now worked also mean a larger administrative block. Besides this the hospital cannot remain the only one of its size without a laundry; on the present washing bill nearly £2,000 is being spent. Toward the total cost of £50,000 five colliery owners have promised to give £11,375 to the fund if other employers will subscribe on a similar scale. The former have also agreed to contribute to the maintenance one-third of the annual sum given by the miners. There are about 45,000 miners in the district, and a meeting of their representatives is recommending the men to increase their contribution from 1½*d.* to 2½*d.* per week. But the miners form only part of the patients, and of the rest many are "unattached" and need the help of the charitable. So far the total promised, including the conditional grant from the Red Cross, is over £24,000. To put in so much good work before the opening meeting promises well for the success of the appeal.

### AMALGAMATION SUGGESTED AT WEYMOUTH.

The Council of the Weymouth and Dorset County Royal Eye Infirmary is considering the suggestion made at the recent representative meeting at Dorchester that the hospitals in Weymouth, in order to prevent overlapping, should be amalgamated. If any scheme of amalgamation is decided upon there are several reasons for continuing to work the Eye Infirmary under the same roof and with the same staff as at present. The existing buildings of the Eye Infirmary, which was established in 1836, comprise two male wards, with six and seven beds respectively; a female ward of seven beds, with two set apart for cataract cases; and an operating-theatre, have done much useful work. During the past ten years 1,616 in-patients and 8,429 out-patients have been treated, and the institution is in the happy position of being able to carry on its work without accumulating a load of debt. Under its present medical officers and the recently appointed matron, Miss Kingham, from Cheltenham, Weymouth Eye Infirmary has a bright prospect before it. As a rule difficulties in the way of amalgamation are varied and complex. Co-ordination is not open to these objections, and has the advantage of linking more institutions than any amalgamation scheme can usually touch.

### INFANT MORTALITY IN HUNGARY.

The accompanying chart, which illustrates in a remarkably clear way the effect of the recent years of war upon the infant mortality in Budapest, was specially compiled for a contributor to The Hospital by Professor Bokay, whose name as the most distinguished physician in Hungary is well-known the world over. The curves can be consulted together or individually. In either case they are decidedly instructive. The mortality and birth curves show how, up to the declaration of war, the number of births had been practically the same for a number of years, whereas the mortality among these children was steadily increasing. In 1914, almost immediately there was a very marked rise in the infantile death-rate and combined at the same time with the inevitable fall in the number of children born, the curves give a striking picture of the effects of many privations and disease.

### THE NATIONAL PHYSICAL LABORATORY.

The recently issued report of the above laboratory contains records of several pieces of work of direct interest to the medical and institutional world. There are a host of details bearing on war material which were not, for obvious reasons, made public in earlier issues, but it is possible here to touch only upon a few outstanding features. A great deal of progress has been made in more accurately standardising the small clinical thermometer. A number of problems dealing with the optical abilities and disabilities of glass were also investigated with most satisfactory results. It should indeed follow that the manufacture of special lenses, etc., will rapidly become a home industry. Radium-testing has received considerable attention, and it is particularly interesting to note that preparation is now being made for a complete investigation of the absorptive qualities of materials used as protectors in $x$-ray work. There have undoubtedly been unnecessary martyrs in this branch of medical work, and the protection provided is certainly in part imperfect at present.

### AN INQUIRY AT PLYMOUTH.

At a recent meeting of the Plymouth Town Council a long debate took place on a communication from the Ministry of Health commenting adversely on the handling by the Medical Officer of Health of three recent fatal cases of puerperal fever in an institution there. The Maternity Committee of the Council had presented a report to the Council, which the latter did not adopt: the Ministry concurred with the Maternity Committee, not with the Council. It was at first proposed that the Council should adhere to its previous resolution (of confidence in the Medical Officer of Health), but this was defeated by a narrow majority, and eventually it was resolved to invite the Ministry to conduct the suggested inquiry. Since the action of the Medical Officer of Health has been publicly impugned, it is by far the best thing both for him and for the town to have an open and impartial inquiry held: meanwhile, it is important to suspend judgment on the whole unfortunate incident at the Three Towns Nursing Home..

### A SHOP FUND AT OXFORD?

Confronted with a deficit and an expensive scheme of extension, the Committee of the Radcliffe Infirmary, Oxford, is issuing two special appeals to the workers and to the villages. The latter suggests that a Hospital Aid Committee should be formed in each village to gather subscriptions from those not subscribing through any other channel. The villages should certainly do their share, since they send more patients than their subscriptions warrant. But the same must be true of the city, and surely more could be done than at present by means of an organisation in the retail shops. Since the wages of the rank and file of the retail trade are smaller than those of other industrial classes, it becomes important that the shopkeepers should set the example to their salesmen. We hope, therefore, that a committee of retailers may be formed. If they will guarantee a subscription per head of their employees the latter will hardly fail to follow suit, and the population of the city is sufficient for a large sum to be raised by these means. We live in an age when large sums are made out of a multitude of pennies, and since a shop is, in the legal sense, a factory, why should not the workers' plan of weekly contributions be extended to all "behind the counter"?

### SIXPENCE A WEEK FROM DERBY.

The extension of the nurses' home at the Derbyshire Royal Infirmary will soon be ready and shortly opened, and the contract for the new out-patients' department has been signed. While the ordinary expenditure has increased by nearly £10,000 for the nine months ending June 30, when compared with the corresponding figure for last year, a welcome example has been set by the Borough Police of the city, who have agreed to subscribe sixpence a week to the infirmary. We hope that their generosity will not be lost upon other bodies. For if the financial status of policemen be compared with that of industrial workers, it will be seen that many of the latter are as well or better off, and yet the police have the honour of this generous example. They deserve every praise for it. Is it too much to hope that the police in other districts may take a similar lead?

### SURGERY OF THE HEART.

The opening of the surgical discussions in Paris to which we recently referred has given us at least one remarkably valuable illlustration of progress. We refer to the question of cardiac surgery dealt with by that distinguished surgeon, Dr. Tuffier of Paris. To the professional surgeon the chief novelty of the address and following discussions lay in the novelties of technique described, but to the unspecialised many of the points made will come as a complete revolution of the accepted teachings of the past. The fact was strikingly shown that cardiac areas in which wounds and injuries have been considered as almost invariably fatal are just those which provide the greatest opportunity of surgical repair. The valves and the impulse conducting paths (*e.g.*, the bundle of His) maintain their position as danger zones, but the musculature of the heart can be repaired with complete success. Dr. Tuffier actually presented details of a series of 305 cases of cardiac injuries, out of which no fewer than 151 recovered after surgical measures had been applied.

### A HOSPITAL REOPENS ITS WARDS.

We are glad to learn that, thanks to an emergency grant of £4,000 from King Edward's Hospital Fund for London, and an extension of the principle of payments by patients, the National Hospital for the Paralysed and Epileptic, Queen Square, W.C., is now re-opening its doors so far as the greater part of the work of the institution is concerned. The Board of Management, however, regrets that three wards will have to remain unoccupied until the receipt of increased monetary assistance. We hope that an energetic and systematic effort on the part of the management will be made to attract fresh subscribers, and that the required increased income may be thus secured.

### THIS WEEK'S DRUG MARKET.

A slight improvement in the tone of business is noticeable, but it is difficult to say whether it will be maintained. The improvement may be due to the fact that some buyers who had been holding off have been compelled to replenish their stocks. There has been a better demand for menthol and the value has moved upwards. Cloves have also made a good recovery from the recent depression. In some of the synthetic drugs, prices are more or less nominal, and offers at below market quotations do not seem to tempt buyers on any considerable scale. Potassium bromide is in plentiful supply and buyers continue to hold aloof. Citric and tartaric acids are quiet, with easier price tendencies in each case. Almond oil is rather cheaper

## Figures and Tables

**Figure f1:**